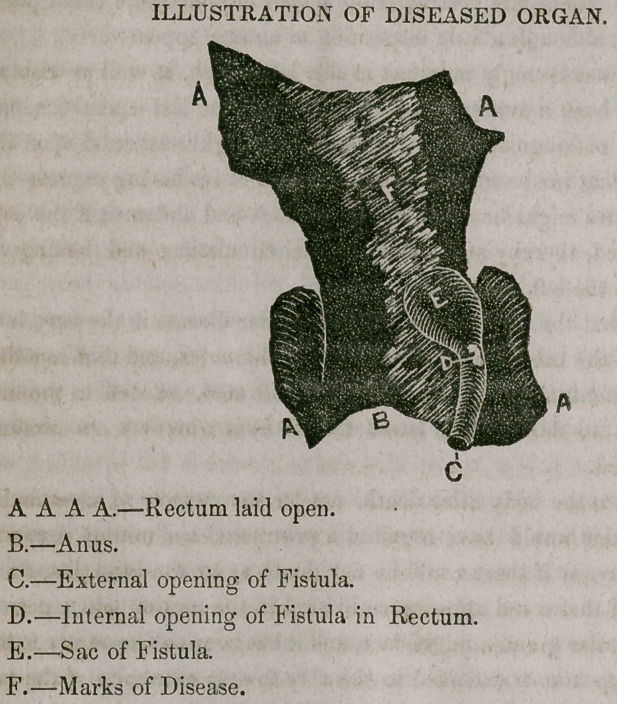# Tetanus Produced by the Application of Nitrate of Silver to Diseased Rectum

**Published:** 1849-08

**Authors:** John K. Hardenbrook

**Affiliations:** Rochester


ART. III.—*Tetanus produced by the application of Nitrate of Silver to diseased rectum*, by JOHN K. HARDENBROOK, M. D.

[We deem it proper to state that, suspicions having been entertained of death having been produced in the following case, by the administration of strychnia with an intent to kill, the reporter of the case was indicted and tried on the charge of murder. The trial resulted in his acquital. We conceive that there can be no impropriety in this statement, inasmuch as the fact, of course, is one of public notoriety, and, probably, has fallen under the notice of most of our readers. Of the circumstances upon which the accusation was based, we know but little, not having taken any pains to obtain information respecting them. We do not allude to the subject in consequence of any opinion we have been led to entertain respecting the case, either in a moral or scientific point of view, but, on the contrary, to disclaim entirely all knowledge or prepossessions with reference to its merits. The report has been sent to us with a request to insert it in our columns, which we do not feel at liberty to decline; but it has seemed to us due, alike to the writer and reader, that the fact which has been mentioned should be premised, since no allusion is made to it in the report itself.—*Editor.*]

While science is daily revealing truths heretofore obscured, and establishing facts, in nature and philosophy, upon incontrovertible bases; while we are enabled, from analogy, to draw rational conclusions, and almost relieved from any hypothetical necessity to enforce our individual views and opinions, it is not a little surprising that the subject matter we are about to record, although known for nearly two thousand years, and having engaged the attention of the most eminent pathologists of all ages, still remains but partially developed as to its nature and specific character.

If we confide at all in nosoligical arrangement, we must class this among neuralgic diseases, and, in the outset, we meet with the most formidable obstacle; for, owing to the minute and delicate structure of the nervous system, pathological enquiries most frequently fail to discover any alteration in its structure, even when unequivocal symptoms point directly to it as the seat of disease, and, moreover, so multiplied and varied in their form and character are many affections attributed to this class, and so frequently are they allied to, and combined with each other, that no little embarrassment arises to the practitioner, in attempting to define, with any degree of positive certainty, the case before him. I will not be understood to say that there are no instances which are not unequivocal, but I affirm that the majority, by far, are so complicated, as to defy a correct diagnosis. It will be found upon reference to "Tweedie," one of the highest authorities upon nervous diseases, that affections of the brain, spinal cord, delirium tremens, epilepsy, apoplexy, chorea, hysteria, hydrophobia, and tetanus, are all, under certain circumstances, marked by precisely the same symptoms, in a greater or less degree, and sometimes blended together; and that thirst, perspiration, vomiting, rigidity, convulsions, consciousness, and the reverse, attend each, and, for this reason, give rise to no trifling difficulty in some cases, to determine which peculiar name shall designate the disease. It is not uncommon for persons unacquainted with these diseases, to give a pretty accurate account, and, at the same time, to omit some very decided symptoms, which the practical eye of the medical observer would alone detect, and give a different feature to his diagnosis from the combined appearances.

The premonitory symptoms I am about to enumerate, will manifest their peculiarity more particularly to the medical reader, and if their striking characteristics did not attract our attention at the time, as being the precursors of a formidable disease, it can only be attributed to the fact, that its prevalence is not so frequent as might reasonably be expected under such vicissitudes of temperature as we live in, and that it most commonly is consecutive to external wounds, while, at the same time, it is engendered by constitutional effects, remaining latent, to be developed by the most trivial exciting cause.

It will not, then, be expected that I shall here submit any theory of this disease, but give a brief account of symptoms as they were observed. This patient, in May, 1848, while absent from his home, was attacked by some disease which his medical attendant informed him was hemorrhoids, or piles, and he was doubtless relieved by his administrations, as nothing was heard of it for some weeks, when he submitted his case, and I inferred from his relation that it must have been as stated. The usual remedies, however, did not appear to fulfil the expected results, for, in connection with a feeling of weight and uneasy sensation, dysenteric symptoms appeared, attended with serious prostration, so as to compel him to go to the country, which he did twice within some fourteen days; and when I visited him, the remedies used at that time appeared efficacious in removing the dysenteric affection, and in greatly relieving the other, at least so far as to be of but little trouble. Sometime in August he again complained of his former disease, with weight and heaviness, and now attended with an ichorous discharge, which produced such extensive excoriation of the perineum, and surrounding parts, as to be painful both in walking or in sitting down, requiring additional clothing to protect him, as well as frequent bathing to secure any degree of comfort. Varied lotions were used, which, for a time, would be efficacious, and promised encouragement of a successful cure, while tonics were used to protect him from prostration. A short time, however, would elapse before a return, with some variation in symptoms. During this whole period, several examinations were made up to within three weeks of his decease; no external disease was manifest, but the most extreme sensitiveness and pain upon introduction of the finger, and examination by instrumental means was entirely out of the question, as a speculum could by no means be introduced. During a period long before his decease, and which I was not aware of until within a short time previously, it had been observed by his friends that a sort of apathy controlled him, and he frequently made incoherent answers to business questions in his store, as well as recently in his family, and would only attend to business with any spirit when his energies were aroused. He had for many weeks complained of head-ache, as if the top was coming off, with redness of eyes, and, at the same time, tongue rather clean, great lassitude, pain from præcordia to back, frequent startings as if from a sciatic affection, restless and uneasy during evenings, which were always spent at home. From being a very sound sleeper, he complained of wakefulness, with frequent violent startings from his sleep; his strength was prostrated, and although he visited his store daily, he could not engage in his usual business to any extent. His appetite had also failed, or was rather very variable. After helping the family he would commence eating, and, before finishing a meal, he would suddenly close his teeth and eat no more. From being a very sedate man, he would suddenly start up, indulge in romping wildly around, and as quickly cease from it. He would sometimes throw his limbs over a low back chair, and whirl around at a rapid rate, laughing excessively. He would be desirous to engage in some evening pastime, and suddenly leave before completing, throw himself on a sofa, restless for a while, and then again resume his play. He would lie upon the sofa for a while, start up with a loud yawn, to the surprise of every one, and rush into a cold north room, declaring he should roast, while no increased temperature of his skin would be perceptible. It seemed, too, for a man of his natural courage, that he suffered under an undue degree of fear; a neighbor's house having been entered by burglars, he constantly talked of it, so much so as to excite terror in the whole family, and frequently exacting a promise that assistance should be rendered upon the least alarm.

For a few days previous to his attack of convulsions, he had been at home, complaining of pain, stiff neck, and sore throat, supposed to be from cold which was, in a measure, relieved by medicine, and he felt as if a short walk would be agreeable, but if he extended his exercise beyond a prudent limit, he returned fatigued, and required his head to be held and bathed for some time. It was noticed at the evening meal, that he took nothing but a cup of tea. In the evening he solicited a game of chequers, and left in the middle of the play, throwing himself on the sofa, as he had done often before. I supposed he had been entertained long enough, but suddenly, after a time, he replaced the men and played again. This was not especially regarded, and was attributed either to satiety or whim; but the various appearances as described, did not escape my notice, and attention was recalled to a previons idea, that some constitutional derangement gave rise to these symptoms; yet nothing was clearly developed from which to form any diagnosis, and occasionally an entire remission of them would occur, which caused some doubt as to the correctness of my suspicions.

Subsequent to these symptoms was an attack of convulsions, the accession of which seems to have been from a sleep upon a lounge, and from which the patient had fallen. Uttering a noise he was found insensible, lying upon his face, with his head resting near the stove upon a spitoon. Being raised up, in a few minutes he set upon the sofa in a state of bewilderment, agitation and trembling, greatly disposed to fall into fits resembling epilepsy. This was soon followed by violent convulsive action, closing the teeth firmly, with countenance somewhat suffused, disposition to draw the knees towards the abdomen, unless held down, which continued for some minutes. This violent attack was succeeded by the spasmodic subsultus frequently observed arising after the convulsions of dentition.

The first idea suggested, was to provide against congestion by copious bleeding, and then to excite vomiting and give an anti-spasmodic. Antimony and morphia being the only remedies at hand, were administered as soon after the bleeding as practicable, and with favorable results, since but one paroxysm succeeded, and the patient felt relieved for some fourteen hours, with the exception of the subsultus or twitching, which continued throughout the succeeding day, rousing him frequently from his sleep. During the convulsions, he had profuse perspiration, which was augmented by having on all his clothes, attended by thirst, after abatement of paroxysms, which will not be regarded as extraordinary, considering the free diaphoresis, and which symptoms have always appeared in the many cases I have witnessed of convulsive diseases. The patient appeared on the following day in rather a favorable condition, with the exception of the subsultus, some pain in the vertebral column, and a great degree of fear lest he might be attacked while sleeping. Every expedient was employed with remedial agents also, to dissipate this fear, and it was confidently hoped, that if he could obtain a few hours' sleep, all apprehension of a return of the convulsions, which were not yet entirely relieved, would cease. Under this encouragement, and the effects of anodynes, he did rest, and slept for nearly two hours before his last attack, when he awoke with an uneasy sensation, which was allowed to exist some time before I was called, and after a short period a violent convulsion ensued, which was in a measure relieved by bleeding, but was soon succeeded by a second, more violent than the others, and destroyed life before any medicine could possibly afford relief, although a little disposition to nausea appeared.

Opisthotonos was strongly manifest in this last attack, as well as trismus, and it has even been a matter of question whether the last convulsion, and its effects, were not augmented by the immense weight sustained upon the body of the patient inadvertently, in consequence of his having expressed a wish that pressure might be made upon the chest and abdomen if the convulsions returned, thereby suspending arterial circulation, and forcing venous blood into the left ventricle.

I have said that the patient was suffering under disease in the very lowest intestine, of the most sensitive and painful character, and that constitutional derangement, although not positively confirmed, existed to produce this, as well as to develop the latent tetanic form wherever an excitant might be applied.

In looking into the body after death, not for the purpose of ascertaining the cause (for this would have required a protracted and minute examination) but to discover if there could be any doubt as to the local disease, it was ascertained that a red appearance existed in the portion taken out, as well as an irregular granulating surface, and it has been subsequently found, that this red appearance extended to the very lowest extremity of the rectum, and that a fistula with a small external, and an internal opening, existed, communicating with a sac one inch each way in diameter, as will be seen in the plate. This doubtless contained the fluid so frequently discharged, and so ichorous in its character, which had its specific effect upon the mucous membrane lining this organ, and which produced the ulcerated appearance already mentioned. With regard to the existing cause, I deemed it proper, as the former remedies had become inert, to use an application of the solution of crystals of Nitrate of Silver, which was injected by the patient himself, and which, beyond doubt, in my mind, was the exciting cause of this formidable disease. The peculiar characteristics before enumerated manifested the agency of a constitutional derangement, which was tending toward the same result, and which might, of itself, in the lapse of a few hours, have developed the disease. I am led to the conclusion, from observation and subsequent reflection, that this was a case similar to the few on record of Idiopathic Tetanus of an Epileptiform character in its accession, and developed by the application of caustic to an ulcerated surface, and I desire to record this, if any benefit may ensue to my professional brethren, who may, like myself, be too prone to give publicity to those cases only, in which their treatment has been eminently successful.

## Figures and Tables

**Figure f1:**